# Comment on “Giant bullous emphysema mistaken for traumatic pneumothorax: A fatal case of pneumothorax” and role of the extended Focused Assessment with Sonography in Trauma (eFAST)

**DOI:** 10.1016/j.ijscr.2019.06.048

**Published:** 2019-06-28

**Authors:** C.M.I. Quarato, M.G. Tinti, M. Sperandeo

**Affiliations:** a Institute of Respiratory Disease, Department of Medical and Surgical Sciences, University of Foggia, Foggia, Italy; b Unit of Geriatric, IRCCS Fondazione Casa Sollievo della Sofferenza, San Giovanni Rotondo, Italy; c Unit of Interventional and Diagnostic, Ultrasound of Internal Medicine IRCCS Fondazione Casa Sollievo della Sofferenza, San Giovanni Rotondo, Italy

We read with great interest the article by Edson et al. [1]. where some important lessons can be learned. The authors described the pitfalls encountered behind the trauma assessment of a patient with a bilateral giant bullous emphysema (GBE) highlighting the importance and the continuous need, especially in the emergency department, of a comprehensive radiologic assessment in order to avoid physicians fatal errors.

The absence of breath sounds, beside the concomitant presence of symptomatic chest pain, hypoxia and respiratory distress ideally require a detailed but also fast clinical assessment, before proceeding with invasive measure, such as chest tube placement as in the case described [1]. Indeed, the clinical picture of GBE may mimic and overlap with pneumothorax’s one, and a correct differential diagnosis imply the need of fast but also adequate radiologic assessment.

On this issue, currently there is a particular emphasis on the role of extended Focused Assessment with Sonography in Trauma (eFAST), and more specifically, on the role of transthoracic ultrasound (TUS) as a fast and valuable tool for pneumothorax detection. Indeed, in the last 20 years, the relative reproducibility of bedside TUS examination allowed the development of several protocols in the emergency settings with the aim to fast ‘answer’ to clinical doubts when present.

In such regard, the most helpful ultrasound (US) findings in the demonstration of pneumothorax are the absence of the “lung sliding” sign, followed by the detection of the “barcode sign” in M-mode, and the absence of US ring-down artifacts (B-lines). Instead, the so called “lung point” may be used to estimate the size of a pneumothorax [2].

It has been shown that TUS negative predictive value in the detection of pneumothorax reach almost 99%, as the presence of the “sliding sign”, recognizable in real time by using the B-mode or time-motion (M-mode), excludes the possibility of this condition in the 70% of the pleural surface visible by ultrasound [2] (Fig. 1). Beside this, its positive predictive value is reported to vary between 55% and 90% [3]. Indeed, the absence of the “sliding sign” and the presence of the “barcode sign” are not a definitive clues, being also found in severe pulmonary fibrosis, fibrothorax, phrenic nerve paralysis, bullae, subpleural cystic mass, panlobular emphysema, in patients with a thoracotomy drainage tube, in cancer invading the chest wall, in pleurisy sequelae, in pulmonary contusion and in other conditions including GBE [3,4].

**Fig. 1:**
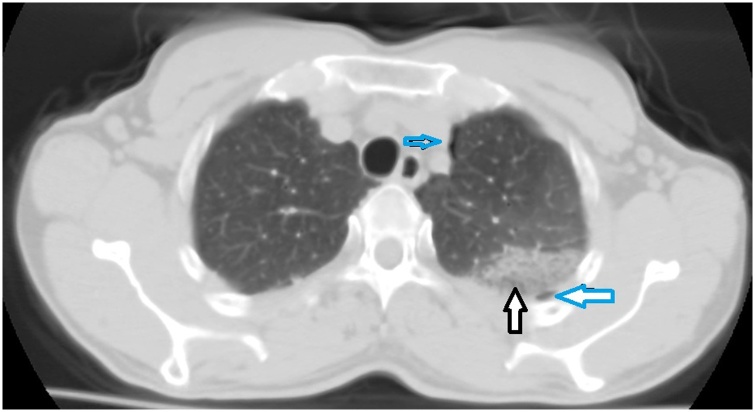
CT axial scan in post traumatic patient with pulmonary contusion (black arrow) and a little pneumothorax (blue arrows), not visible with transthoracic ultrasound, because they are located in areas not accessible to ultrasound (behind the scapula and in the mediastinal pleura).

This occurs because in the normal aerated lung the hyperechoic “pleural line”, that does not correspond anatomically to the parietal or visceral pleura and nor to the pleural space (being the whole pleural space of maximum 150 μm in thickness), but appears to be an interface generated by the elevated difference in acoustic impedance between the chest wall and the pulmonary air, is visualized in real time by B-mode as an horizontal “to-and-fro movement” synchronized with respiratory excursions. This sign is also called “gliding” or “sliding sign”. In M-mode, that detects motion over time, the sliding sign is recognizable as a granular pattern (“the sand”) under the horizontal motionless layers of the chest wall, resulting in the so called “seashore sign”. All conditions that prevent hyperechoic pleural lines’ movement, such as the presence of air in the pneumothorax or any mechanical or physical hindrance of the interface, delete the sliding movement, causing the absence of the classic gliding or sliding sign typical of the normally ventilated lung. Similarly, the M-mode trace in a condition of pneumothorax, but also in all its false positive conditions, will only display a pattern of parallel horizontal lines above and below the pleural line, exemplifying the lack of movement (i.e. showing a “barcode sign”).

These data were also confirmed in a recent review by Richards JR et al. in which eFAST has shown an imperfect sensitivity and specificity for the detection of pneumothorax in presence of underlying pulmonary diseases [5].

Nevertheless, LUS cannot detect pneumothoraxes located in areas not accessible to ultrasound, such as areas hidden by the bony structures of the rib cage and the mediastinal area [[6], [7], [8], [9], [10], [11]].

Therefore, even in the reported case of GBE, the urgent support of ultrasound would have only confirmed the clinical false suspicion, not avoiding the dramatic consequences of an invasive management (i.e. the insertion of bilateral chest tubes) without waiting for radiologic confirmation.

## Funding

We have not any sources of funding to declare, including any study sponsors in the collection, analysis and interpretation of data, in the writing of the manuscript and in the decision to submit the manuscript for publication

## Ethical approval

The manuscript submitted is a simply comment on an article not involving any studies on patients or volunteers and therefore requiring any ethical approval.

## Consent

The manuscript submitted is a simply comment on an article published on this journal, not involving any studies on patients or volunteers and therefore requiring any consent.

## Author contribution

- Carla Maria Irene Quarato, MD, corresponding author, writing - review & editing, Institute of Respiratory Disease, Department of Medical and Surgical Sciences, University of Foggia, Foggia, Italy, c.quarato@libero.it.
- Maria Giulia Tinti, MD, writing - review & editing, Unit of Geriatric IRCCS Fondazione Casa Sollievo della Sofferenza, San Giovanni Rotondo, Italy. mariagiulia.tinti@gmail.com.
- Marco Sperandeo, MD, writing - review & editing, Unit of Interventional and Diagnostic Ultrasound of Internal Medicine IRCCS Fondazione Casa Sollievo della Sofferenza, San Giovanni Rotondo, Italy. sperandeom@libero.it.

## Registration of research studies

The manuscript submitted is a simply comment on an article published on this journal, not involving any studies on human participants.

## Guarantor

Carla Maria Irene Quarato, MD, corresponding author, Institute of Respiratory Disease, Department of Medical and Surgical Sciences, University of Foggia, Foggia, Italy, c.quarato@libero.it

Maria Giulia Tinti, MD, Unit of Geriatric IRCCS Fondazione Casa Sollievo della Sofferenza, San Giovanni Rotondo, Italy. mariagiulia.tinti@gmail.com

Marco Sperandeo, MD, Unit of Interventional and Diagnostic Ultrasound of Internal Medicine IRCCS Fondazione Casa Sollievo della Sofferenza, San Giovanni Rotondo, Italy. sperandeom@libero.it

## Declaration of Competing Interest

We have not any actual or potential conflict of interest to disclose, including any financial, personal or other relationships with other people or organizations that could inappropriately influence or bias our work.

## CRediT authorship contribution statement

**C.M.I. Quarato:** Writing - review & editing. **M.G. Tinti:** Writing - review & editing. **M. Sperandeo:** Writing - review & editing.
